# A framework for understanding mast cell activation syndrome from a complex adaptive systems perspective

**DOI:** 10.3389/fsysb.2026.1980056

**Published:** 2026-10-30

**Authors:** Werner Richardt Van Zyl, Sanchen Henning

**Affiliations:** 1 Independent Researcher, Johannesburg, South Africa; 2 University of South Africa School of Business Leadership (SBL), Pretoria, South Africa

**Keywords:** complex adaptive systems, feedback dynamics, mast cell activation syndrome, neuroimmune interaction, systems biology

## Abstract

Mast Cell Activation Syndrome (MCAS) challenges conventional reductionist medical models because patients experience fluctuating symptoms across autonomic, immune, gastrointestinal, and behavioural domains, with substantial inter-individual variability and often only partial responses to organ-specific interventions. This conceptual approach reframes MCAS as a complex adaptive system in which chronic illness emerges from interacting feedback dynamics spanning the nervous, immune, behavioural, and environmental domains, rather than from isolated dysfunction within a single subsystem. The framework identifies two primary reinforcing feedback structures: a nervous system loop characterised by autonomic and stress-related dysregulation, and an immune system loop characterised by escalating inflammatory activity. Their persistent coupling generates higher-order emergent dynamics, including amplification, persistence, and instability, which are moderated by balancing regulatory processes that support adaptive stability. This systems perspective addresses fluctuating symptoms, multisystem involvement, and variable treatment responses commonly observed in MCAS. From this perspective, health reflects the capacity to maintain functional adaptation under perturbation, whereas disease emerges when dynamics exceed the system’s regulatory capacity. The framework is proposed as a hypothesis-generating scaffold that complements established biomedical approaches and seeks to stimulate future empirical research into the systems-level dynamics of chronic multisystem illness.

## Introduction

Although modern medicine has succeeded in diagnosing and treating diseases through linear causal identification, a reductionist approach often overlooks the dynamic interactions, interdependencies, and nonlinear relationships that characterise living systems. Many chronic diseases remain classified as idiopathic or of unknown cause, with fluctuating symptoms, multisystem involvement, and substantial variation among individuals (O'Connor et al., 2006).

Patients with MCAS often present with fluctuating, multisystem symptoms affecting autonomic, immune, gastrointestinal, and other physiological domains (Molderings and Afrin, 2023). These complexities challenge conventional biomedical models that rely on linear causal relationships within isolated physiological systems. This conceptual framework highlights the dynamic interactions among neurological, immunological, behavioural, and environmental processes that influence health outcomes over time. Chronic illness is an emergent property of multiple interconnected, self-reinforcing processes operating across multiple levels of the organism.

This perspective does not replace the reductionist approach that underpins much of modern medicine but complements it by providing a systems-level explanation of conditions that are difficult to understand with linear models alone. CAS theory emphasises feedback loops, nonlinearity, adaptation, and shifts in system stability (Preiser, 2024; Rizi, 2026). Within this framework, MCAS is both a clinical condition and a useful case study for broader questions about chronic disease, adaptive regulation, and systemic health.

## Theoretical background

Complex adaptive systems (CAS) consist of multiple interacting components whose collective behaviour cannot be fully understood by studying individual parts alone (Lansing, 2003; Tukamuhabwa et al., 2015; Long et al., 2018; Sturmberg et al., 2019; Artime et al., 2024). CAS are adaptive and nonlinear, exhibiting emergent system-level interactions among components (Preiser, 2024). Five concepts from complexity science underpin the proposed framework: regulatory feedback loops, homeostasis, emergence, nonlinear dynamics, and paradox.

Living systems function through the interplay of feedback loops. Positive feedback amplifies change and promotes divergence within a system, whereas negative feedback counteracts change and supports adaptive regulation and stability (Allen and Malekpour, 2023). The balance between these opposing processes determines whether a system remains resilient or moves towards dysregulation. At one extreme, rigid equilibrium signifies the absence of adaptive capacity and is associated with illness or death; at the other extreme, uncontrolled instability likewise threatens system viability. Health is therefore best understood as a state of organised disequilibrium, characterised by continual adaptation and a dynamic balance between these extremes (Prigogine and Nicolis, 1985).

Homeostasis is not a static equilibrium but an ongoing process of adaptive regulation (Billman, 2020; El-Samad, 2021; Lloyd et al., 2001). Disease can be understood as a dynamic state in which regulatory capacity progressively diminishes, producing increasingly rigid, self-reinforcing patterns of behaviour (Wichers et al., 2019). In this sense, pathology emerges when a system becomes so trapped in maladaptive regulatory dynamics that it can no longer counterbalance effectively.

Emergence refers to system-level properties that arise from interactions among components rather than from components acting in isolation (Rizi, 2026). From this perspective, chronic illness is understood as an emergent property of interactions across neurological, immunological, behavioural, and environmental domains, rather than the result of a single pathological mechanism (Olthof et al., 2023). Disease and ill-health emerge when reinforcing dynamics increasingly exceed the system’s capacity for adaptive regulation (Dai et al., 2012).

Nonlinear dynamics indicate that biological responses are not proportional to their causes. Small changes can produce substantial effects once activation thresholds are crossed, whereas large interventions may have limited impact if the underlying regulatory state remains unchanged (Peters et al., 2004; Scheffer et al., 2012). Cumulative subthreshold stressors may progressively lower these thresholds until a minor trigger precipitates significant escalation, while delays between perturbation, mediator release, and symptom manifestation displace cause from effect. These dynamics help explain why patients with similar diagnoses often experience markedly different symptom trajectories and treatment responses.

These concepts operate across nested levels: molecular/cellular (mediator release, cytokine signalling), physiological (autonomic and inflammatory activity), behavioural/psychological (stress appraisal, coping, sleep), and environmental/contextual (allergens, infection, life stressors). The system boundary is defined by this coupled neuroimmune–behavioural–environmental network; comorbidities or processes outside this coupling fall outside the framework’s explanatory scope.

Because outcomes emerge from nonlinear interactions, a treatment effective in one context can worsen symptoms in another. Such apparently paradoxical responses may reflect differences or shifts in system regulatory state rather than simple intervention-response relationships (Hayes and Andrews, 2020; Strunk and Lichtwarck-Aschoff, 2019). Together, these five concepts form the theoretical foundation for the conceptual framework in Figure 1.

**FIGURE 1:**
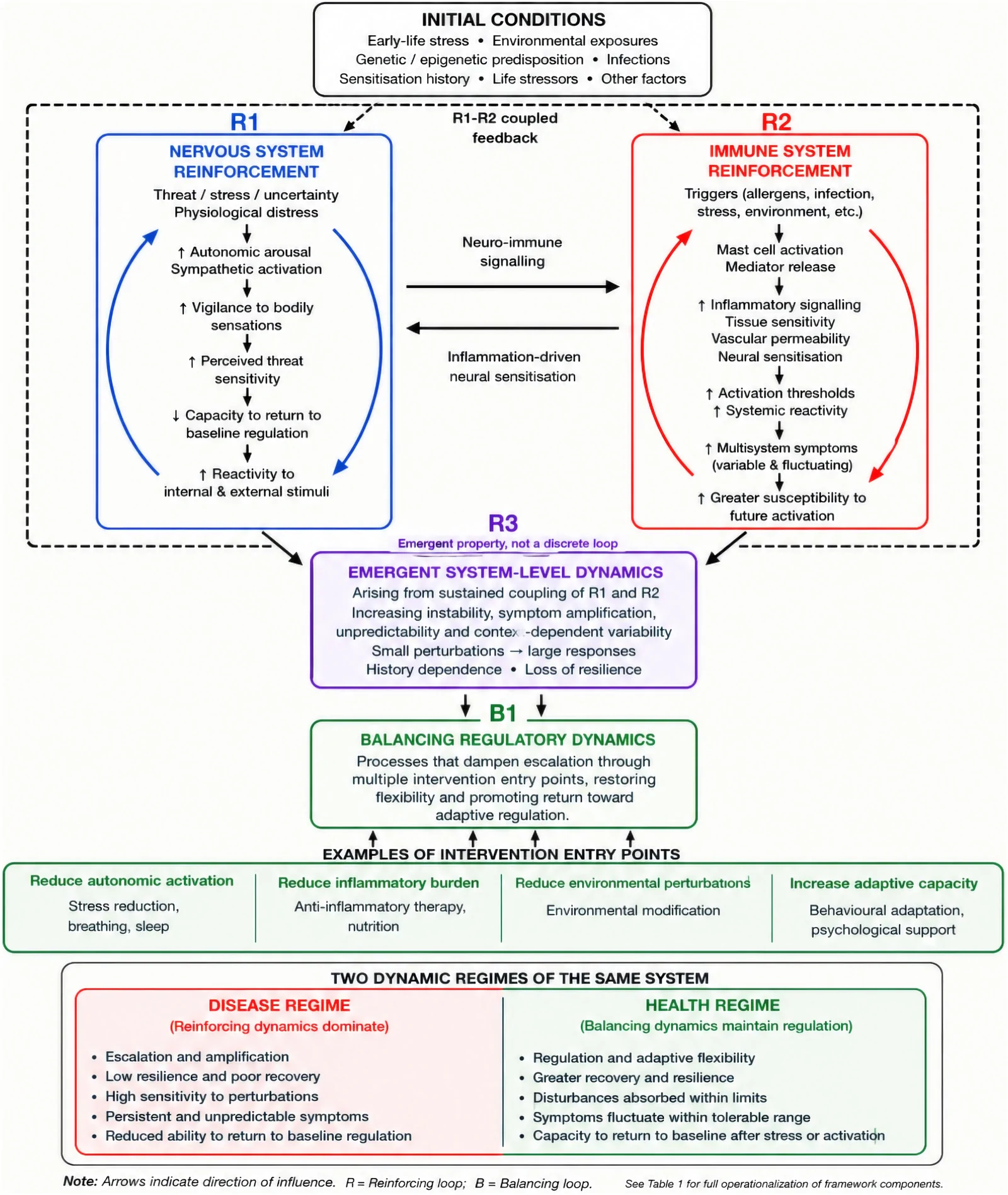
The Mast Cell Complex Adaptive Systems (MCCAS) framework of neuroimmune dysregulation. Reinforcing nervous system feedback dynamics (R1) and immune system feedback dynamics (R2) interact through bidirectional neuroimmune signalling, producing higher-order emergent instability (R3). R3 indicates an emergent property, not a discrete reinforcing loop. Balancing regulatory dynamics (B1) represent regulatory processes that may attenuate escalation and promote system stabilisation. Initial conditions influence system susceptibility and activation thresholds.

## Illustrative patient narrative on diagnosed mast cell activation syndrome

The following vignette is intended to demonstrate the framework’s explanatory potential and should not be construed as evidence of its validity or as proof of the underlying disease mechanisms. It illustrates how a systems-oriented perspective may help to organise and interpret the evolution of complex chronic illness over time.

The patient is a 40-year-old South African man diagnosed with MCAS and with a history of fluctuating multisystem symptoms spanning more than 3 decades. These symptoms span gastrointestinal, dermatological, autonomic, cognitive, and muscoskeletal domains, with marked variability in severity. Symptoms often shift between organ systems and are frequently disproportionate to objective clinical findings, making them difficult to accommodate within the boundaries of individual medical specialties. An internal medicine specialist diagnosed MCAS based on a multisystem symptom pattern, food intolerances, response to a low-histamine diet (despite normal serum tryptase), after excluding alternative diagnoses (Van Zyl, 2024).

Viewed through the lens of the proposed framework (Figure 1), this trajectory reflects the intensification of reinforcing dynamics within the nervous system (R1) and the immune system (R2). These factors appear to interact recursively rather than independently, contributing to heightened system sensitivity and nonlinear responses to minor perturbations. Continued reciprocal reinforcement between neural (R1) and immune (R2) processes is understood to give rise to the emergent system-level instability represented by R3 in Figure 1. Rather than constituting a distinct pathological process, R3 signifies a higher-order emergent property of the coupled system that arises when reinforcement overwhelms regulation.

When interpreted through the lens of the above proposed framework, as the inflammatory burden gradually declines through the combined effects of complementary interventions, autonomic regulation is consistent with becoming more effective This pattern suggests that the efficacy of individual interventions may depend less on their isolated effects than on the overall regulatory state of the coupled neuroimmune system. Collectively, these interventions may be interpreted as strengthening the balancing dynamics (B1) by acting at multiple levels within the regulatory network.

Small perturbations sometimes produced disproportionately large symptom escalations (Gómez-Carrillo and Kirmayer, 2023; Scheffer et al., 2024), whereas narrowly targeted, symptom-focused interventions yielded only partial or temporary improvements.

Paradoxically, attempts to regulate autonomic activation during periods of acute inflammatory escalation did not initially improve overall system stability and, at times, even worsened symptoms.

From this perspective, wellness and stabilisation are not merely the absence of symptoms but rather reflect the system’s flexibility, reduced reactivity to stress, and its capacity to return to a balanced regulatory state (Bettinger and Friston, 2023; Sturmberg, 2021).

## The mast cell complex adaptive systems (MCCAS) framework

The proposed MCCAS framework is presented as a conceptual, hypothesis-generating diagram rather than a definitive account of disease mechanisms. It provides a systems-oriented perspective for interpreting symptom dynamics, disease persistence, and therapeutic responses in complex chronic illness. Thus, the framework has not yet been empirically validated, and the proposed causal relationships, feedback structures, system states, indicators and interventions therefore require longitudinal empirical testing.

The framework comprises two interacting regulatory domains: the nervous and immune systems. Interacting neuroimmune processes may contribute to the persistence, amplification, variability, and regulatory instability associated with MCAS.

### Initial conditions and system predisposition

The trajectory of MCAS is shaped by its initial conditions, including early-life stress, environmental exposures such as trauma, and genetic predisposition, which may lower activation thresholds and increase susceptibility to dysregulation (Balouek et al., 2023; Mogil, 2021). Figure 1 highlights two major reinforcing feedback processes that may underlie the persistence and amplification of symptoms in MCAS: the nervous system loop (R1, left) and the immune system loop (R2, right). Although depicted as distinct loops for explanatory purposes, they function as tightly coupled components of a larger regulatory network, exerting continuous reciprocal influence on one another. The interaction of these reinforcing processes may contribute to the emergence and maintenance of chronic dysregulation at the system level.

### R1 dynamics of nervous system reinforcement

The first reinforcing process involves autonomic and stress-related dysregulation within the nervous system. In this loop, threat, uncertainty, physiological distress, and chronic stress activation may increase autonomic arousal and sympathetic nervous system activity (Matyevich et al., 2025; Shao et al., 2023). Increased arousal may subsequently heighten alertness to bodily sensations (Zhang et al., 2024), heighten perceived threat sensitivity (Richter et al., 2021), and diminish the system’s capacity to return to baseline regulation after activation (Herhaus et al., 2026). These effects further reinforce autonomic activation, creating a self-amplifying feedback loop. Over time, repeated cycling through this loop may gradually sensitise the nervous system to both internal and external stimuli, thereby increasing the likelihood of persistent dysregulation and symptom amplification.

### R2 dynamics of immune system reinforcement

The second reinforcing process involves escalation of immune activation. Mast cell degranulation and mediator release heighten tissue sensitivity, vascular permeability, neural sensitisation, and systemic reactivity (Miller et al., 2021; Nguyen et al., 2021). Intensified signalling produces variable, oscillatory symptoms across organs, while ongoing activation amplifies instability and sustains mast cell activity.

From a systems perspective, symptoms that appear unrelated at the clinical level may represent different manifestations of the same underlying regulatory dynamics (Hayes and Andrews, 2020; Sturmberg et al., 2019; Sturmberg, 2021). Fluctuating symptoms may arise from a single interconnected system operating across multiple levels, rather than from separate diseases, thereby contributing to the heterogeneous and dynamic clinical presentation characteristic of MCAS.

Although R1 and R2 are depicted as distinct reinforcing processes, the framework proposes that persistent dysregulation emerges from their mutual feedback rather than from isolated functioning.

## Cross-system coupling and emergent dynamics in R3

Chronic symptom patterns are the emergent behaviour of a tightly interconnected neuroimmune system in which reinforcing feedback operates at multiple levels of organisation (Koban et al., 2021; Plum et al., 2024; Xu et al., 2020).

R3 represents a higher-order emergent dynamic arising from sustained coupling between R1 and R2 (Figure 1). This dynamic can produce variable symptoms, greater unpredictability, and heightened sensitivity to previously tolerated stimuli (Andersen et al., 2023; Jahanbani et al., 2024). In the MCAS framework, mast cell activation and mediator release drive changes in vascular permeability, tissue sensitivity, and neural signalling across multiple organs (Miller et al., 2021; Nguyen et al., 2021). Thus, it is not a generic model of chronic neuroimmune dysfunction but a conceptual model that explains MCAS through the interaction between mast cell-mediated immune processes and nervous system regulation. R3 is not a third reinforcing loop with independent causal closure but an emergent, system-level property arising from sustained R1–R2 coupling. It is therefore observable only through its downstream effects (variability, instability, sensitisation) rather than as a discrete mechanism in its own right.

MCAS provides the biological context for conceptualising R2 dynamics, while the emergent R3 dynamics may be examined through observable changes in symptom burden, symptom variability, trigger sensitivity, and, where clinically appropriate and validated, biomarkers of mast cell activation and inflammatory activity. As an emergent system-level phenomenon, R3 is not directly observable but may be inferred from patterns of behaviour exhibited by the system over time.

Potential indicators of system-level behaviour include changes in symptom burden, volatility, number of systems affected, frequency and severity of exacerbations, shifts in trigger sensitivity, and recovery time after perturbation. Mast cell-associated biomarkers such as N-methylhistamine, leukotrienes, and prostaglandins may add insight, especially when tracked longitudinally across symptomatic and stable periods.

## Dynamics and regulation intervention points balancing (B1)

Biological systems contain balancing processes that counteract reinforcing dynamics and support adaptive regulation. In Figure 1, these processes are represented by B1. Their function is not to eliminate variability, which is inherent in living systems, but to prevent reinforcing feedback from overwhelming the organism’s regulatory capacity.

B1 does not denote a single treatment or mechanism but rather a class of regulatory processes that operate at multiple points in the system. Different individuals may achieve greater stability through different intervention pathways, depending on which dynamics predominantly contribute to dysregulation. Potential intervention points include autonomic regulation, reducing inflammatory burden, treating persistent infection, environmental modification, restoring sleep, and behavioural adaptation. Rather than a universal cause, the framework highlights diverse high-impact intervention points within a broader network, with therapeutic gains arising from strengthening balancing dynamics across the system.

A systems-theoretical perspective reframes symptoms as expressions of the organism’s current regulatory state. Changes in symptom severity, variability, or sensitivity may reflect shifts in underlying system dynamics, including autonomic activation, inflammatory burden, and environmental exposures. From this perspective, symptoms are observable signals of ongoing regulatory activity within the larger system.

Within the proposed framework, improvement is not necessarily defined by the complete elimination of symptoms. Instead, it may be reflected in reduced symptom volatility, lower sensitivity to perturbations, faster recovery from challenges, and greater overall system flexibility. These changes would be consistent with a shift in the balance between reinforcing and balancing dynamics towards a more stable, adaptive regulatory state.

## Dynamic regimes: pathology and health in the same system

In a pathological regulatory regime, reinforcing processes such as chronic autonomic activation and escalating inflammation may amplify one another (Koban et al., 2021; Androulakis, 2024). As coupling intensifies, system sensitivity increases, activation thresholds decrease, and recovery time after perturbation lengthens. The organism becomes less able to absorb disturbances without destabilisation (Forzieri et al., 2022), leading to symptoms that are persistent, unpredictable, and spread across multiple domains.

In a healthier, adaptive regulatory system, balancing processes are more effective at curbing escalation and restoring flexibility after activation. The system continues to fluctuate within safe limits, preventing sustained runaway activation. Health is therefore defined not by symptom absence but by regulatory capacity—the ability to adapt, recover, and return toward a viable dynamic regime after perturbation.

Disease, in contrast, may arise when reinforcing dynamics progressively overwhelm the system’s regulatory capacity, shifting the organism towards an increasingly unstable and less recoverable state. From this perspective, the distinction between health and disease is not categorical but dynamic, reflecting the system’s evolving position along a continuum of regulatory stability and instability.

Complexity theorists argue that adaptive functioning is often not in perfect balance but rather in a dynamic region between excessive order and excessive instability: a continuum of Stable equilibrium, Bounded instability, and Explosive instability (Henning, 2009). This perspective frames health and wellness as a state of Bounded instability, a midpoint within which variation, adaptation, and novelty occur within functional limits. Both excessive Stable equilibrium (rigid stability) and Explosive instability (runaway instability) are maladaptive (Ciaunica et al., 2023).

The proposed framework portrays health and disease as regulatory processes defined by the balance between reinforcing and stabilising dynamics within the system (Cohen et al., 2022; Merabet et al., 2022). These insights help explain why chronic illness often fluctuates over time (Craddock et al., 2014) and why relatively small changes in one domain can sometimes produce disproportionately large effects elsewhere in the organism (Sturmberg, 2021).

## Towards operationalising the MCCAS framework

Building on the central hypothesis, three sub-propositions follow. P1: Sustained bidirectional coupling between R1 and R2 precedes measurable R3 instability, such that tracking autonomic and inflammatory markers simultaneously should show increasing correlation before symptom escalation. P2: Strengthening B1 processes reduces symptom volatility and reduces recovery time independent of which specific intervention point is targeted. P3: Individuals in a disease regime show disproportionate (nonlinear) symptom responses to standardised perturbations compared to those in a health regime. Table 1 translates these propositions into observable indicators, expected dynamic behaviour, and study designs capable of testing them empirically.

**TABLE 1:** Operationalising the MCCAS framework.

Component	Observable indicators	Expected dynamic behaviour	Study approach
R1 (nervous system reinforcement)	Heart rate variability, autonomic reactivity, self-reported vigilance/threat sensitivity	Reduced capacity to return to baseline after activation	Repeated measures of autonomic testing across symptomatic/stable periods
R2 (immune system reinforcement)	N-methylhistamine, leukotrienes, prostaglandins, symptom burden by organ system	Escalating reactivity to previously tolerated triggers	Longitudinal biomarker tracking alongside symptom diaries
R3 (emergent instability)	Symptom volatility, number of systems affected, exacerbation frequency/severity, trigger sensitivity	Amplified, unpredictable responses to minor perturbations	Time-series analysis of co-occurring R1/R2 indicators
B1 (balancing dynamics)	Recovery time after perturbation, symptom fluctuation range	Faster return to baseline, reduced volatility	Before-and-after tracking of self-regulation of the autonomic and immune systems restoring homeostasis in trials
Disease vs. health regime	Composite of above across time	Persistent instability vs. bounded, recoverable fluctuation	Cohort comparison using regime-classification criteria

## Discussion

The hypothesis developed for further empirical research is:

In individuals with MCAS, persistent dysregulated interactions between nervous and immune processes may contribute to reduced physiological resilience and adaptive capacity, thereby promoting the persistence, amplification and variability of mast-cell activation, mediator release and associated symptoms.

Unlike multisystem disease models that classify affected organs, a systems approach treats feedback, emergence, nonlinearity, and regulatory capacity themselves as the object of study, explaining not just where dysfunction occurs but how it is sustained.

This perspective may extend beyond MCAS to other complex chronic conditions (e.g., chronic pain, autonomic disorders, post-viral syndromes) that share feedback-driven amplification and persistent dysregulation despite differing biological mechanisms (Thurner and Stengel, 2023; Davis et al., 2023).

Patients with these conditions are often managed within organ-based or specialty-specific models of care (Eyowas et al., 2023). Although these approaches remain essential for identifying and treating local pathology, they may be less effective when clinical outcomes arise from interactions across multiple regulatory domains (Ouellet et al., 2024).

Figure 1 outlines a systems-based framework that complements existing approaches to symptom fluctuation, variable treatment responses, and system sensitivity. Rather than replacing biomedical investigation or disease-specific care, it highlights changes in broader regulatory states to help clinicians and patients interpret multisystem illness within a coherent model (Hodgson et al., 2024; You et al., 2025).

Rather than focusing solely on symptom suppression, clinicians can identify and target key regulatory processes within the broader system (Martyushev-Poklad et al., 2022). These leverage points vary across patients: for some, environmental exposures drive instability, whereas for others, autonomic dysregulation, inflammation, persistent infection, or chronic stress are the primary drivers of system behaviour.

Although conceptual in nature, as with the illustrative narrative above, these applications remain hypothesis-generating rather than clinically validated, yet may help bridge the biological, behavioural, and environmental dimensions of chronic illness within a unified systems model.

## Conclusion

The authors present MCAS as a complex adaptive system in which illness stems not from a single fault but from dynamic feedback across neurological, immunological, behavioural, and environmental domains. Dysregulation arises through reinforcing feedback loops, while recovery restores balancing mechanisms that support resilience across interconnected systems. This perspective helps explain why patients with complex chronic conditions often face fragmented care.

Building on this view, the authors call for a paradigm shift from asking where disease resides to how dysregulation emerges across biological, behavioural, and environmental systems. The MCAS framework applies complexity science to chronic illness, suggesting that persistent symptoms arise from dynamic interactions rather than isolated pathological processes.

## Data Availability

The original contributions presented in the study are included in the article/supplementary material, further inquiries can be directed to the corresponding author.
